# Chronic psychosocial stress disturbs long-bone growth in adolescent mice

**DOI:** 10.1242/dmm.030916

**Published:** 2017-12-01

**Authors:** Sandra Foertsch, Melanie Haffner-Luntzer, Jochen Kroner, Florian Gross, Kathrin Kaiser, Maike Erber, Stefan O. Reber, Anita Ignatius

**Affiliations:** 1 Laboratory for Molecular Psychosomatics, Clinic for Psychosomatic Medicine and Psychotherapy, University, 89081 Ulm, Germany; 2 Institute of Orthopedic Research and Biomechanics, University Medical Center Ulm, 89081 Ulm, Germany

**Keywords:** Chronic psychosocial stress, Bone, Growth, Chronic subordinate colony housing

## Abstract

Although a strong association between psychiatric and somatic disorders is generally accepted, little is known regarding the interrelationship between mental and skeletal health. Although depressive disorders have been shown to be strongly associated with osteoporosis and increased fracture risk, evidence from post-traumatic stress disorder (PTSD) patients is less consistent. Therefore, the present study investigated the influence of chronic psychosocial stress on bone using a well-established murine model for PTSD. C57BL/6N mice (7 weeks old) were subjected to chronic subordinate colony housing (CSC) for 19 days, whereas control mice were singly housed. Anxiety-related behavior was assessed in the open-field/novel-object test, after which the mice were euthanized to assess endocrine and bone parameters. CSC mice exhibited increased anxiety-related behavior in the open-field/novel-object test, increased adrenal and decreased thymus weights, and unaffected plasma morning corticosterone. Microcomputed tomography and histomorphometrical analyses revealed significantly reduced tibia and femur lengths, increased growth-plate thickness and reduced mineral deposition at the growth plate, suggesting disturbed endochondral ossification during long-bone growth. This was associated with reduced Runx2 expression in hypertrophic chondrocytes in the growth plate. Trabecular thicknesses and bone mineral density were significantly increased in CSC compared to singly housed mice. Tyrosine hydroxylase expression was increased in bone marrow cells located at the growth plates of CSC mice, implying that local adrenergic signaling might be involved in the effects of CSC on the skeletal phenotype. In conclusion, chronic psychosocial stress negatively impacts endochondral ossification in the growth plate, affecting both longitudinal and appositional bone growth in adolescent mice.

## Abstract (precis)

**Summary:** Chronic psychosocial stress is a known risk factor for many somatic disorders. The authors demonstrate that chronic psychosocial stress negatively affects endochondral ossification in the growth plate, as well as long-bone growth. This article has an associated First Person interview with the first author of the paper as part of the supplementary information.

## INTRODUCTION

Chronic psychosocial stress represents an increasing burden in our modern society and is an accepted risk factor for the development of numerous mental disorders, including post-traumatic stress disorder (PTSD) and depression (Yehuda and Seckl, 2011; Gold et al., 1988). Both diseases display a high prevalence in western countries and are strongly comorbid with various somatic pathologies (Gebara et al., 2014; Glaesmer et al., 2012). Both PTSD and depression were indicated to be associated with osteoporosis and increased bone fracture risk in a number of studies (Glaesmer et al., 2012, 2011; Calarge et al., 2014; Gebara et al., 2014; Zong et al., 2016). However, although there is strong evidence for an increased risk for low bone mass and fragility fractures in depressed patients, possibly because of increased levels of glucocorticoids (GCs) (Cizza et al., 2001, 2010), findings in PTSD patients are less consistent. For example, multivariable analyses controlling for depression in PTSD subjects failed to reveal a link between PTSD and osteoporosis (Tsai and Shen, 2017), whereas earlier studies stated a significant association between these conditions (Glaesmer et al., 2011). Furthermore, PTSD may influence long-bone growth: children subjected to repeated mental traumatization during childhood were of a significantly shorter stature (Batty et al., 2009). In summary, these clinical studies implicate different effects of stress-induced depression and PTSD on bone turnover.

Preclinical studies revealed reduced bone mass in murine models of chronic mild stress, in which mice were subjected to a series of mild and unpredictable physical and/or psychological stressors, resulting in a depressive-like phenotype (Liu and Liu, 2017), basal hypercorticism and sympathetic nervous system (SNS) activation (Azuma et al., 2015; Furuzawa et al., 2014; Yirmiya et al., 2006; Baldock et al., 2014). However, these models might have limitations in trying to reflect the situation in PTSD patients, who suffer from hypo- rather than hypercorticism (Yehuda and Seckl, 2011). In contrast to the chronic mild-stress model, the chronic subordinate colony housing (CSC) paradigm, a validated mouse model for social-stress-associated PTSD (Reber et al., 2016), only causes chronic activation of the SNS but not of the stress-associated hypothalamic-pituitary-adrenal (HPA) axis (Reber et al., 2007). This is indicated by increased plasma norepinephrine (NE; noradrenaline) concentrations (Reber et al., 2007), basal evening hypocorticism and unaffected basal morning plasma corticosterone (CORT) concentrations following 19 days of CSC exposure (Langgartner et al., 2017; Reber et al., 2007). Moreover, in contrast to the chronic mild-stress model, CSC mice display increased anxiety-related behavior without displaying concomitant changes in depressive-like behavior (Slattery et al., 2012). CSC mice further show a reduced *in vitro* adrenocorticotropic hormone (ACTH) sensitivity of adrenal explants (Uschold-Schmidt et al., 2012), increased adrenal and decreased thymus weight (Reber et al., 2007), and increased myelopoiesis in the bone marrow (Schmidt et al., 2016), as well as local and systemic immune activation (Schmidt et al., 2016; Reber, 2014; Reber et al., 2011, 2007; Schmidt et al., 2010). Given the striking differences between the CSC paradigm and other chronic stress models in terms of behavioral and physiological effects, we hypothesize that the CSC-induced PTSD phenotype does not induce a decrease in bone mass but rather affects bone length. Consistent with this hypothesis, rats exposed to 4-7 weeks of psychosocial isolation stress during rearing displayed increased bone mass and mineralization (Schiavone et al., 2016). Therefore, in the current study, we investigated whether or not the CSC paradigm affects bone homeostasis and growth in adolescent mice, and how this might be mechanistically mediated.

## RESULTS

### CSC increased anxiety-related behavior and induced typical signs of chronic psychosocial stress

**Fig. 1.**
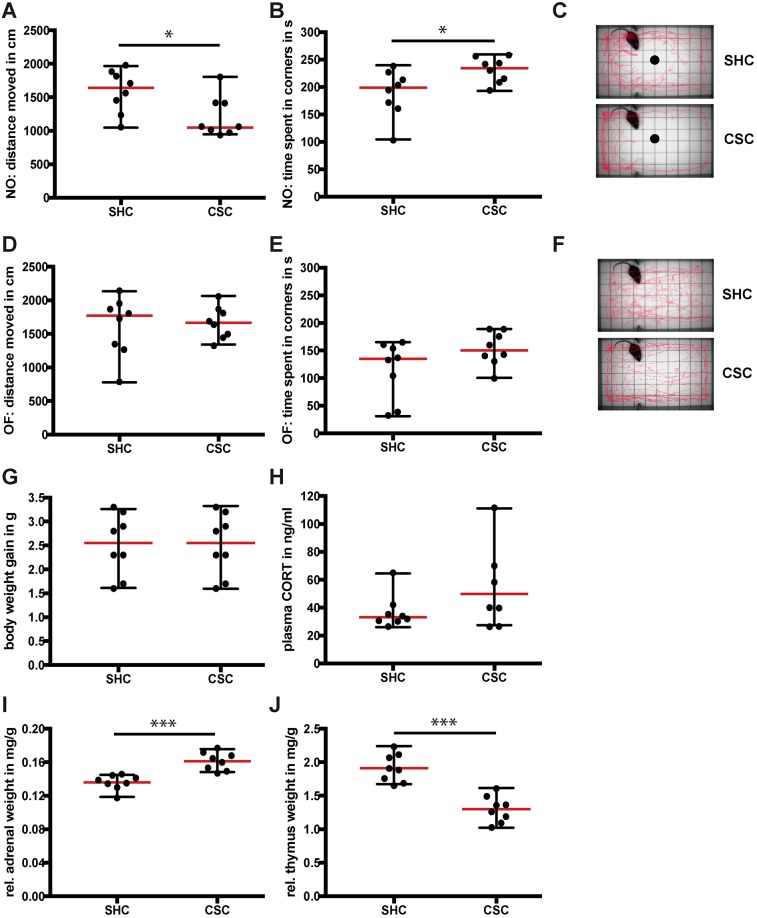
**Behavioral and physiological parameters following 19 days of chronic subordinate colony housing (CSC).** (A) Distance moved during 5 min of novel object (NO) exposure, (B) time spent in corners of the arena during 5 min of NO exposure, and (C) representative track of locomotor activity of a single-housed colony control (SHC) and a CSC mouse during 5 min of NO exposure. (D) Distance moved during 5 min of open field (OF) exposure, (E) time spent in the corners of the arena during 5 min of OF exposure and (F) representative track of locomotor activity of an SHC and a CSC mouse during 5 min of OF exposure. (G) Body-weight gain during 19 days (day 20 measurement – day 1 measurement) of CSC exposure, and (H) plasma corticosterone (CORT) concentrations, (I) relative adrenal weight and (J) relative thymus weight following 19 days of CSC. Data are displayed as individual dot plots with median (red)±range (black). *0.05≥*P*≥0.01; ***P<0.001.

CSC mice displayed increased anxiety-related behavior in the open-field/novel-object (OF/NO) test on day 14 of CSC exposure (Fig. 1A-F). This was indicated by a decreased distance moved (Fig. 1A) and increased time spent in the corners of the arena (Fig. 1B) during NO exposure in CSC compared with single-housed for control (SHC) mice. An unaffected distance moved during OF exposure (Fig. 1D) indicated that locomotion in general was unaltered by CSC. The time spent in the corners of the arena (Fig. 1E) during OF exposure also did not differ significantly between SHC and CSC mice. Body-weight gain (Fig. 1G) and plasma morning CORT concentrations (Fig. 1H) did not differ between SHC and CSC mice, whereas the relative adrenal weight was significantly increased (Fig. 1I) and the relative thymus weight was significantly decreased (Fig. 1J) in CSC compared to SHC mice, indicating enhanced SNS activation.

### CSC altered growth-plate architecture and disturbed endochondral ossification

**Fig. 2.**
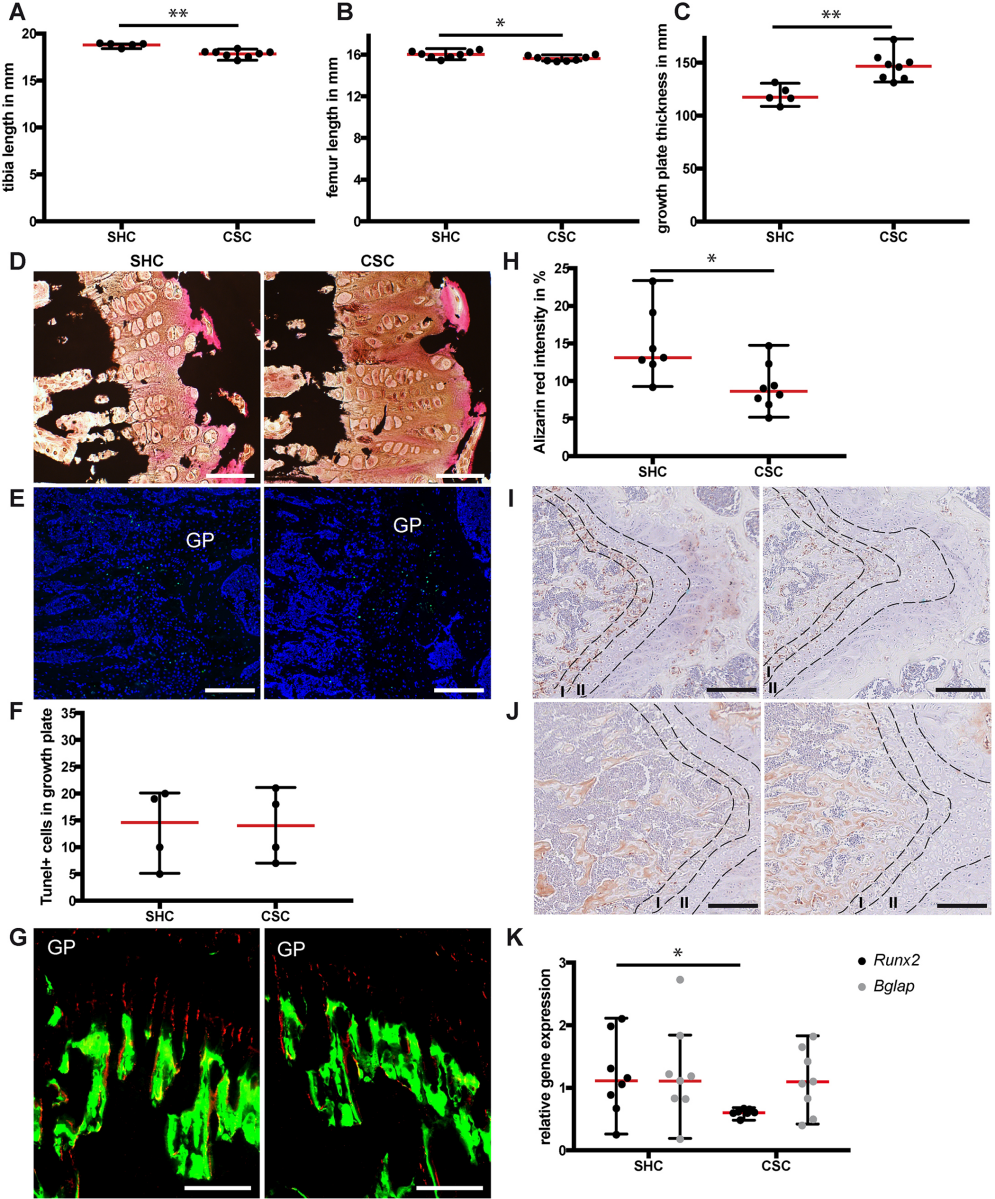
**Longitudinal bone-growth parameters following 19 days of chronic subordinate colony housing (CSC) exposure.** (A) Tibia length, (B) femur length, (C) femoral growth-plate thickness, (D) von Kossa staining of the femoral growth plates (scale bars: 50 µm), (E) TUNEL staining of the femoral growth plates (GP, growth plate; scale bars: 200 µm), (F) quantification of TUNEL-positive cells in the growth plate, (G) fluorescence labels (Calcein Green and Alizarin Red) at the femoral growth plate (scale bars: 200 µm) and (H) quantification of Alizarin Red intensity in percent of whole fluorescence intensity. (I) Runx2 immunostaining of the femoral growth plates (scale bars: 200 µm); I, calcification zone; II, hypertrophic zone. (J) Osteocalcin immunostaining of the femoral growth plates (scale bars: 200 µm). (K) Relative gene expression of *Runx2* and *Bglap* analyzed by qPCR from distal tibia homogenates. Values were normalized to β2 microglobulin (*B2M*); SHC, single-housed control. Data are displayed as individual dot plots with median (red)±range (black). *0.05≥*P*≥0.01, **0.01>*P*>0.001.

Tibia and femur lengths were significantly reduced in CSC versus SHC mice (Fig. 2A,B), whereas femoral growth-plate width was increased following CSC (Fig. 2C). Growth plates of CSC mice displayed a disorganized chondrocyte column morphology accompanied by an enlarged hypertrophic zone (Fig. 2D). CSC mice displayed similar numbers of TUNEL-positive chondrocytes as SHC mice (Fig. 2E,F), indicating that apoptosis was unaffected by CSC. However, decreased Alizarin Red staining was evident, mainly in terminal chondrocytes of the growth plate of CSC versus SHC mice, indicating reduced cartilage mineralization following CSC exposure (Fig. 2G,H). Furthermore, CSC mice displayed decreased Runx2 expression in chondrocytes located in the hypertrophic and calcification zones of the growth plate, indicating disturbed terminal chondrocyte differentiation during endochondral ossification (Fig. 2I). Osteocalcin staining was mainly found in the matrix of the bone trabeculae and in osteoblasts at the metaphysis, and was unaffected by CSC (Fig. 2J). Quantitative real-time PCR (qPCR) analysis of distal tibia homogenates confirmed unaltered osteocalcin expression and significantly decreased Runx2 expression (Fig. 2K).

**Fig. 3.**
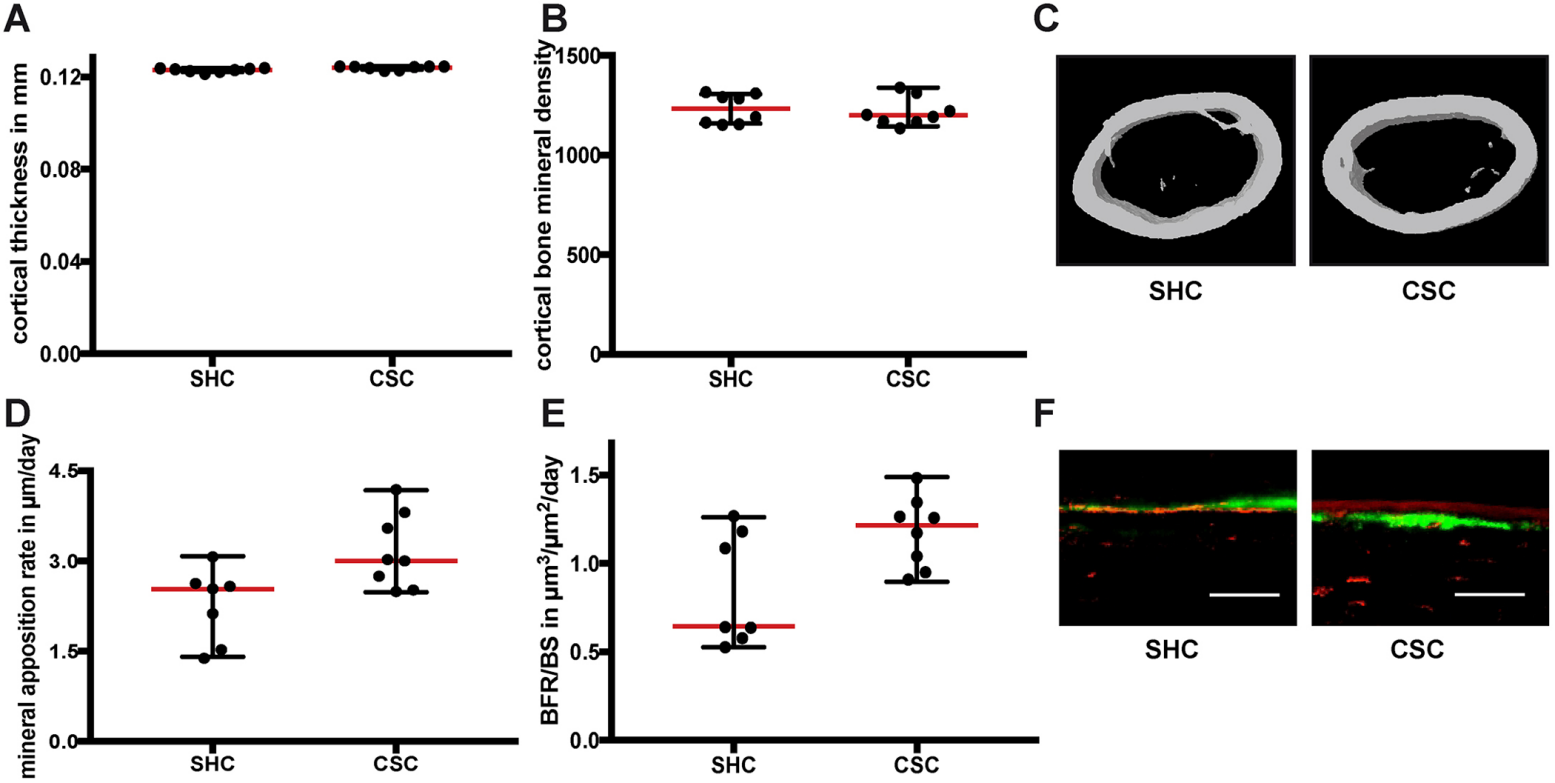
**Cortical bone parameters in the femur following 19 days of chronic subordinate colony housing (CSC).** (A) Cortical thickness, (B) cortical bone mineral density and (C) representative µCT three-dimensional reconstructions of the midshaft of the femur of a single-housed control (SHC) (left panel) and a CSC (right panel) mouse. (D) Periosteal mineral apposition rate, (E) periosteal bone formation rate/bone surface (BFR/BS) and (F) representative images of the fluorescence labels at the midshaft of the femur of a SHC (left panel) and a CSC (right panel) mouse. Scale bars: 50 µm. Data are displayed as individual dot plots with median (red)±range (black).

**Fig. 4.**
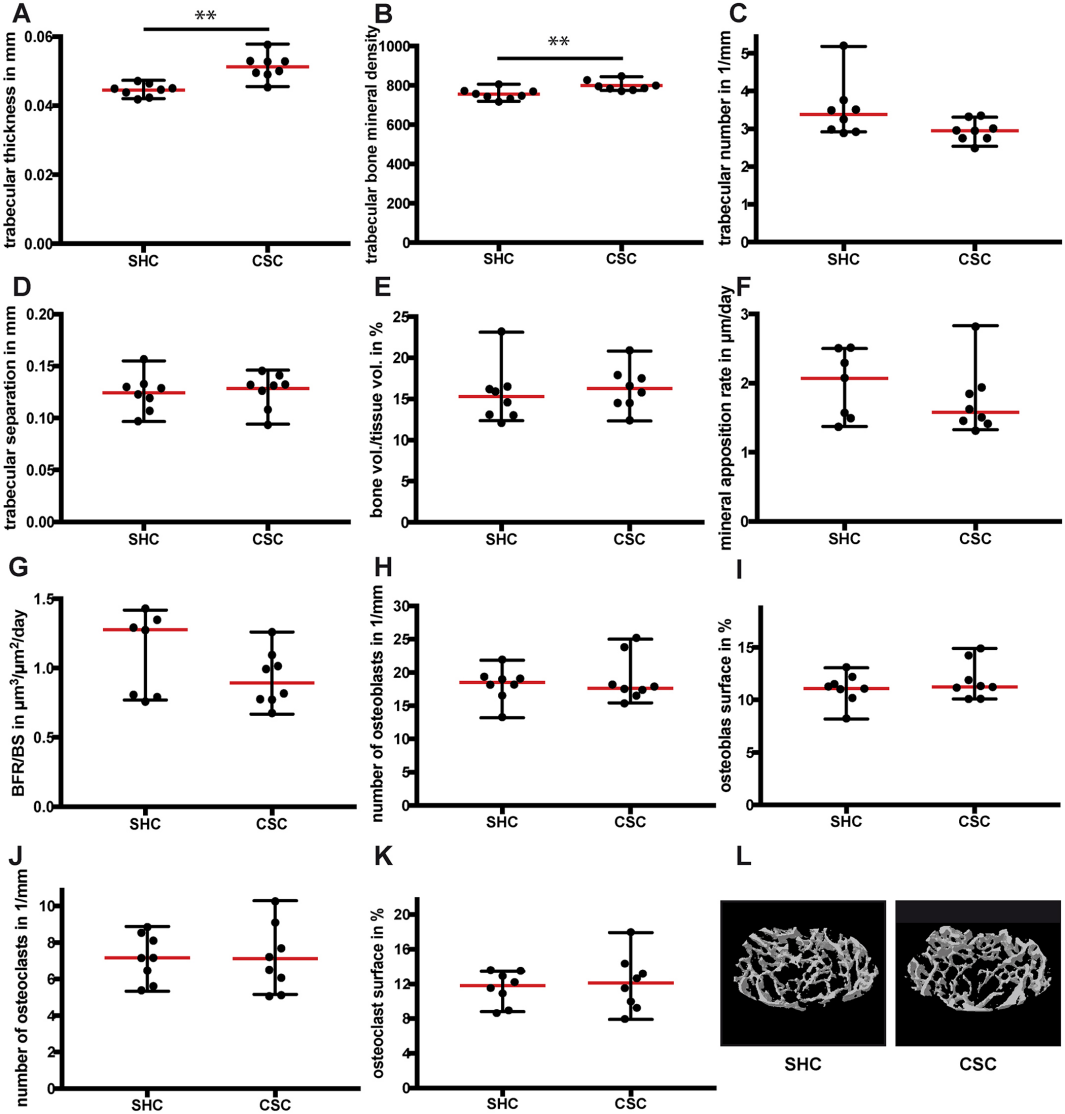
**Trabecular bone parameters in the femur following 19 days of chronic subordinate colony housing (CSC).** (A) Trabecular thickness, (B) trabecular bone mineral density, (C) trabecular number, (D) trabecular separation, (E) bone volume/total volume ratio, (F) mineral apposition rate, (G) bone formation rate/bone surface (BFR/BS), (H) number of osteoblasts per bone perimeter, (I) surface of osteoblasts per bone surface, (J) number of osteoclasts per bone perimeter and (K) surface of osteoclasts per bone surface. (L) Representative µCT three-dimensional reconstructions of the metaphyseal region of the femur. SHC, single-housed control. Data are displayed as individual dot plots with median (red)±range (black). **0.01>*P*>0.001.

Microcomputed tomography (µCT) and histomorphometric analyses of the femurs revealed that CSC mice displayed slightly increased cortical thickness (Fig. 3A,C) because of a slightly increased periosteal mineral apposition rate (Fig. 3D,F). Cortical bone mineral density (BMD) and bone formation rate (BFR) were unaffected (Fig. 3B,E). In the femur metaphysis, CSC mice displayed increased trabecular thickness and BMD (Fig. 4A,B,L), whereas trabecular number, trabecular separation, bone volume ratio, mineral apposition rate and BFR remained unchanged (Fig. 4C-G). The percentage of mineralizing surface [MS; MS/bone surface (BS)] did also not differ between the groups (SHC: 55.6±5.3%; CSC: 54.5±5.3%). Additionally, the number and surface of osteoblasts and osteoclasts were not significantly affected by CSC (Fig. 4H-K). In conclusion, our results demonstrated disturbed endochondral ossification during long-bone growth in CSC mice, which also marginally affected appositional bone growth without any effects on osteoblasts or osteoclasts.

Furthermore, we analyzed mice that underwent 19 days of CSC followed by 21 days of single housing (CSC+SH) to investigate potential prolonged effects of CSC. Control mice were singly housed for 41 days. Length of the femora (Fig. S1A) and trabecular thickness did not differ between the groups (Fig. S1B), whereas trabecular bone mineral density was significantly decreased in CSC+SH mice compared to SHC mice (Fig. S1C). Trabecular number and bone volume to tissue volume ratio were slightly decreased, whereas trabecular separation was slightly increased in CSC+SH mice (Fig. S1D-F). This suggests a negative effect of 19 days of CSC followed by 21 days of single housing on BMD, but no prolonged effects on bone length, possibly due to the relatively short CSC period.

### CSC activated the SNS systemically in the adrenal cells and increased local presence of catecholamine-producing cells in the bone

**Fig. 5.**
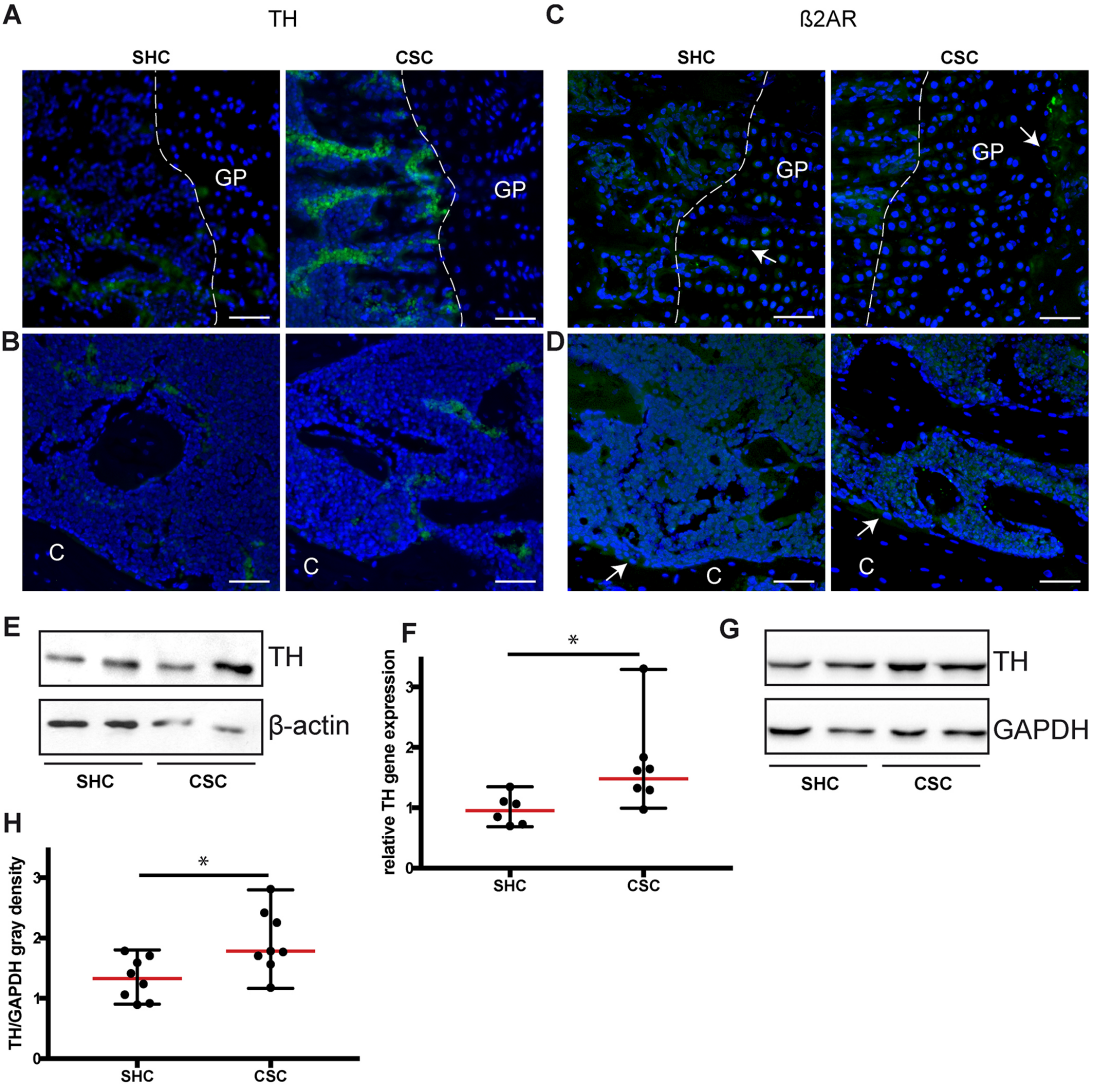
**Sympathetic signaling in the femur and the adrenal gland following 19 days of chronic subordinate colony housing (CSC).** (A) Immunofluorescence staining for tyrosine hydroxylase (TH) at the growth plate (GP) and (B) the metaphyseal region (C, cortex) of the femur. (C) β2-adrenergic receptor (β2AR) staining (arrows) at the GP and (D) the metaphyseal region of the femur. Scale bars: 50 µm, *n*=4-6. (E) Western blot analysis of TH in tibia lysates; β-actin was used as the reference protein. (F) Relative gene expression of *TH* analyzed by qPCR from distal tibia homogenates. Values were normalized to *B2M*. (G,H) Western blot analysis of TH in adrenal gland lysates. Glyceraldehyde 3-phosphate dehydrogenase (GAPDH) was used as the reference protein. SHC, single-housed control. Data are displayed as individual dot plots with median (red)±range (black). *0.05≥*P*≥0.01.

Immunohistochemical staining revealed an elevated number of tyrosine hydroxylase (TH)-positive bone marrow cells at the growth plate of CSC compared to SHC mice (Fig. 5A). In contrast, there were no distinct differences in the metaphyseal region of the femur (Fig. 5B). Moreover, at the epiphyseal growth plate, hypertrophic chondrocytes were found to be β2-adrenoreceptor (β2AR)-positive (arrows in Fig. 5C; Fig. S2A). In the metaphyseal region, most bone marrow cells as well as osteoblasts and osteoclasts (arrows in Fig. 5D; Fig. S2B) displayed β2AR expression. However, there were no obvious differences in β2AR staining between SHC and CSC mice in both compartments. Western blot analysis of whole-tibia lysates (Fig. 5E) and qPCR analysis of distal tibia homogenates (Fig. 5F) confirmed increased local expression of TH in the bones of CSC compared with SHC mice. Higher TH protein expression was further detected in the adrenal glands of CSC versus SHC mice, indicating increased systemic SNS activation (Fig. 5G,H).

## DISCUSSION

The aim of the present study was to investigate the effects of the CSC paradigm, an established mouse model for PTSD, on bone homeostasis and growth in adolescent mice. Our data demonstrated a negative influence of CSC on longitudinal bone growth. The lengths of the long bones were significantly reduced, accompanied by an increased growth-plate width. Decreased Runx2 staining in the chondrocytes of CSC mice at the hypertrophic and mineralization zones of the growth plate as well as altered cartilage mineralization indicated disturbed chondrocyte differentiation during cartilage-to-bone transition. Furthermore, CSC mice displayed increased cortical and trabecular thicknesses, although osteoblast and osteoclast numbers and activities did not differ. This indicates a dysregulation of endochondral bone formation by CSC, affecting both longitudinal and appositional bone growth. Increased TH expression in the adrenal medulla and locally at the growth plate indicated that enhanced catecholamine signaling might play a complex role in mediating CSC effects on endochondral ossification.

In the present study, the OF/NO test was performed to assess anxiogenic effects. Confirming previous data of our group (Peters et al., 2013), CSC mice displayed increased anxiety-related behavior already after 14 days of CSC exposure, indicated by a decrease in the distance moved during NO exposure and an increased time spent in the corners of the arena while the NO was present. The total distance moved during OF exposure was unaffected by CSC, suggesting that general locomotion was unchanged. Additionally, in agreement with our own previous findings (Reber et al., 2007; Langgartner et al., 2015, 2017), CSC mice displayed increased adrenal weight, decreased thymus weight and unaffected plasma morning CORT concentrations compared to SHC mice. Of particular relevance for this study was that body weight was unaffected by CSC exposure, because it is widely accepted that differences in body weight can account for altered bone mass (Nguyen et al., 1998).

Our results indicate that CSC disturbed the endochondral ossification process at the growth plate, possibly mediated by decreased Runx2 expression during chondrocyte differentiation. Runx2 expression is increased in hypertrophic chondrocytes during endochondral ossification (Otto et al., 1997) and plays a crucial role in the ossification process: Runx2-deficient mice display severe limb shortening and enlarged hypertrophic zones in the growth plates (Inada et al., 1999). Confirming this, mice deficient for matrix metalloproteinase 13, a downstream target of Runx2 (Jiménez et al., 1999), show disturbed endochondral ossification in the growth plates, but also thicker cortices and trabeculae and hypermineralized bone (Stickens et al., 2004; Tang et al., 2012). This indicates that dysregulated endochondral ossification might influence appositional bone growth, as we similarly found in the present study.

In contrast to our study, decreased bone mass was reported in mice following exposure to chronic mild stress (Yirmiya et al., 2006; Furuzawa et al., 2014; Azuma et al., 2015; Lertsinthai et al., 2015), an accepted stress model for inducing a depressive-like phenotype and increased plasma GC and NE concentrations. Because GCs are established inhibitors of bone formation by increasing osteoblast and decreasing osteoclast apoptosis (Weinstein et al., 2004, 1998; O'Brien et al., 2004), the difference in the HPA axis activity might contribute to the different effects of CSC and chronic mild stress on bone metabolism and formation. Further support for this hypothesis is provided by Henneicke et al., who showed that chronic mild-stress-induced bone loss was abolished in mice with disrupted GC signaling in osteoblasts and osteocytes (Henneicke et al., 2016). In agreement with this, it was shown that patients suffering from depressive disorders often displayed increased serum GC levels and were at higher risk for developing osteoporosis (Vrkljan et al., 2001). In contrast, PTSD patients suffered from hypo- rather than hypercorticism (Yehuda and Seckl, 2011) and have been shown to develop a shorter stature as young adults when severe traumatization occurred during childhood (Batty et al., 2009). Moreover, multivariable analyses controlling for depression in PTSD subjects failed to reveal a link between PTSD and osteoporosis (Tsai and Shen, 2017).

In addition to GCs, SNS activation might play an important role in mediating stress-induced changes in bone metabolism. Treatment with the β-adrenergic antagonist propranolol reduced chronic mild-stress-induced bone loss in mice (Yirmiya et al., 2006). Furthermore, chronic mild stress increased the proliferation of bone marrow hematopoietic stem cells, which is dependent on β3-adrenergic signaling (Heidt et al., 2014). Because mice subjected to CSC also displayed increased plasma NE levels (Reber et al., 2007), we investigated the systemic activation of the SNS as well as indications for a local activation of the catecholamine-producing machinery in CSC compared with SHC mice. Increased TH expression in the adrenal glands of CSC mice confirmed enhanced systemic SNS activation. Notably, locally in the bone, increased TH expression following CSC exposure was only observed in bone marrow cells in close proximity to the growth plates, indicating a possible influence of locally produced catecholamines on growth-plate chondrocytes. Furthermore, growth-plate chondrocytes were found to express β2AR, which is discussed as the major receptor involved in neuronal control of bone formation (Takeda et al., 2002). However, the influence of SNS neurotransmitters on chondrocyte differentiation, and therefore long-bone growth, is currently not fully understood (Jenei-Lanzl et al., 2014; Lorenz et al., 2016; Lai and Mitchell, 2008; Niedermair et al., 2014). But, indeed, administration of βAR-blockers attenuates growth retardation in severely burned children, who display increased plasma NE levels (Herndon et al., 2012, 2016). Furthermore, it was shown that SNS signaling in osteoblasts via βARs acts catabolically on bone by suppressing bone formation and enhancing bone resorption (Takeda et al., 2002; Takeda and Karsenty, 2008). Therefore, administration of βAR-blockers leads to enhanced bone formation in both mice (Takeda and Karsenty, 2008; Yirmiya et al., 2006; Teong et al., 2017) and humans (Pasco et al., 2004; Toulis et al., 2014). However, because osteoblast numbers and activity did not differ in our study, βAR signaling in osteoblasts may be unaltered following CSC, as indicated by the unchanged TH expression level at the metaphyseal bone region. Further studies using chondrocyte-specific βAR-deficient mice are needed to unravel the underlying mechanism after CSC exposure.

In conclusion, we demonstrated an altered endochondral ossification process during long-bone growth in adolescent mice subjected to a mouse model of PTSD. Decreased chondrogenic differentiation in the growth plate was associated with disturbed cartilage-to-bone transition and cartilage mineralization, whereas osteoblast and osteoclast activities remained unchanged. The skeletal phenotype of CSC mice might be associated with increased systemic SNS activation and a local activation of the catecholamine-producing machinery.

## MATERIALS AND METHODS

### Animals

Male C57BL/6N mice (experimental mice; 19-22 g) and male CD-1 mice (dominant resident mice; 30-35 g) were obtained from Charles River (Sulzfeld, Germany). All mice were kept under standard laboratory conditions (12 h light/12 h dark cycle, lights on at 06:00 am, 22°C, 60% humidity) and had free access to tap water and standard mouse diet.

### Study approval

All animal experiments were in compliance with international regulations for the care and use of laboratory animals (ARRIVE guidelines and EU Directive 2010/63/EU for animal experiments) with the approval of the Local Ethical Committee (No. 1219, Regierungspräsidium Tübingen, Germany).

### Experimental procedures

Seven-week-old mice were either chronically stressed by a 19-day exposure to the CSC paradigm or single-housed for control (SHC). A first set of SHC (*n*=8) and CSC (*n*=8) mice was tested for anxiety-related behavior in the OF/NO test on day 14 of CSC between 07:00 and 10:00 am. Both SHC and CSC mice were injected with the fluorochromes Calcein Green at day 15 and Alizarin Red at day 18 to assess dynamic bone formation. Animals were euthanized on the morning of day 20 between 07:00 and 10:00 am by rapid decapitation following brief CO_2_ anesthesia to assess physiological and endocrine parameters, including plasma CORT, body-weight gain, and relative adrenal gland and thymus weights as well as parameters regarding bone metabolism. Both femurs, tibiae and lumbar vertebral bodies were removed for further analysis. Femur and tibia lengths were assessed using a digital precision caliper. Further, a second set of mice was chronically stressed by a 19-day exposure to the CSC paradigm followed by 21 days of single housing (CSC+SH mice; *n*=8). Control mice (*n*=6) were singly housed for 41 days.

### CSC paradigm

The CSC paradigm was performed as described previously (Reber et al., 2007, 2008; Langgartner et al., 2015). Briefly, after being single housed for 1 week after arrival, experimental CSC mice were housed together with a dominant male CD-1 mouse for 19 consecutive days to induce a stressful situation. To avoid habituation, CSC mice were introduced into the home cage of a novel dominant male mouse on days 8 and 15 of the CSC procedure. Except for a weekly change of bedding, the respective SHC mice remained undisturbed in their home cages for 19 consecutive days. Single housing was demonstrated to be the adequate control group for the CSC paradigm, because group housing itself is able to affect CSC-induced physiological changes (Singewald et al., 2009).

### OF/NO test

The OF/NO test was conducted with SHC and CSC mice on day 14 of the CSC paradigm as previously described (Veenema et al., 2008), with minor modifications. Briefly, the arena (45 cm height×27 cm length×27 cm width) was subdivided into an inner and an outer zone and the four corners. At the start of the first trial, the mouse was placed into the inner zone of the arena and was allowed to explore the arena for 5 min. Immediately after this OF exploration, a plastic round object (diameter: 3.5 cm; height: 1.5 cm) was placed in the middle of the inner zone. During this 5-min trial, the mouse was allowed to explore the arena containing the unfamiliar object. In the OF/NO test, the distance moved and the time the animals spent in the corners were analyzed. Furthermore, in the OF test, the number of inner-zone entries and, in the NO test, the number of object explorations, were analyzed. All parameters were analyzed using EthoVision XT (version 9, Noldus Information Technology, Wageningen, The Netherlands). The test was performed between 08:00 am and 12:00 pm under white light conditions (300 lux).

### Determination of body, adrenal and thymus weights

After decapitation under CO_2_ anesthesia on day 21, the adrenal glands were removed, pruned of fat and weighed. Right adrenal glands were rapidly frozen in liquid nitrogen and stored at −80°C until further analysis. The thymus was removed and stored in ice-cold phosphate-buffered saline until all animals were euthanized. Subsequently, thymus glands were pruned of fat and weighed separately.

### Trunk blood sampling

Within 3 min after removing the cage from the animal room, mice were anesthetized by brief CO_2_ exposure followed by decapitation. Trunk blood was collected in ethylenediaminetetraacetic acid (EDTA)-coated tubes (Sarstedt, Nuembrecht, Germany) and stored on ice until centrifugation. Tubes were centrifuged at 4°C (5000 ***g***, 10 min). Plasma samples were stored at −20°C until further analysis.

### Enzyme-linked immunosorbent assay (ELISA) for plasma CORT

Plasma samples were analyzed using a commercially available ELISA for CORT (analytical sensitivity: <0.564 ng/ml, intra-assay and inter-assay coefficients of variation ≤6.35%; IBL International, Hamburg, Germany).

### µCT analysis

Femurs were fixed in 4% paraformaldehyde. µCT scanning was performed using the SkyScan 1172 (Kontich, Belgium) operating at 50 kV and 200 mA. Voxel resolution was set at 8 µm. Three-dimensional analysis was conducted using CTAn and CTVol software according to ASBMR guidelines (Bouxsein et al., 2010). In the femur, two volumes of interest (VOI) were analyzed: VOI 1 (trabecular bone in the metaphyseal region) was set 200 µm proximal to the metaphyseal growth plate with a length of 280 µm. VOI 2 (cortical bone) covered the area from 80 µm proximal to 80 µm distal from the middle of the diaphysis. BMD was assessed using two phantoms with defined hydroxyapatite (HA) contents (250 and 750 mg/cm^3^). The threshold for mineralized tissue was set at 390 mg HA/cm^3^ for trabecular bone and 642 mg HA/cm^3^ for cortical bone.

### Histomorphometry

After µCT scans, right femurs were subjected to undecalcified histology as described previously (Haffner-Luntzer et al., 2014). Sections of 7 µm were stained with Giemsa, Toluidine Blue, tartrate-resistant acid phosphatase or Von-Kossa to analyze the width of the growth plate, number and surface of osteoblasts, number and surface of osteoclasts and mineralized bone, respectively, as described previously (Haffner-Luntzer et al., 2016). Unstained sections were analyzed for fluorochrome labeling to assess dynamic histomorphometry as described in the ASBMR guidelines (Dempster et al., 2013). Analysis was performed using image-analysis software from Leica (Leica MMAF 1.4.0 Imaging System) or the Osteomeasure system (Osteomeasure). Amount of Alizarin Red deposition in the growth plate was assessed by taking images from the respective region and processing them by image-analysis software (Adobe Photoshop, Adobe Systems, Ireland). The amount of all fluorescent pixels and amount of red pixels was assessed by the color picker tool. The percentage of Alizarin Red intensity to whole fluorescent intensity was calculated.

### Immunohistochemistry, immunofluorescence staining and TUNEL assay

Left femurs were fixed in 4% paraformaldehyde and subjected to decalcified histology as described previously (Haffner-Luntzer et al., 2014). Sections of 7 µm were prepared for immunohistochemical staining. Staining for osteocalcin and Runx2 was performed using the following antibodies: rabbit anti-mouse osteocalcin antibody (orb77048, Biorbyt), rabbit anti-mouse Runx2 antibody (8486, Cell Signaling), goat-anti rabbit IgG-biotin (sc-3840, Santa Cruz) and horseradish peroxidase (HRP)-conjugated streptavidin (Zytomed Systems, Berlin, Germany). 3-amino-9-ethylcarbazol (Zytomed Systems) was used as the chromogen and the sections were counterstained using hematoxylin (Waldeck, Münster, Germany). Immunofluorescence staining for TH and β2AR was performed using the following antibodies: rabbit anti-mouse β2AR antibody (sc-569, Santa Cruz), rabbit-anti mouse TH antibody (AB152, Millipore), goat-anti rabbit IgG-biotin (sc-3840, Santa Cruz) and FITC-streptavidin (40201, BioLegend). Species-specific non-targeting immunoglobulins were used as isotype controls. Apoptosis staining was conducted using the CF488A TUNEL apoptosis detection kit (Biotium, Fremont, USA) according to the manufacturer's protocol.

### Protein extraction and western blotting

For analysis of TH expression, right adrenal glands were homogenized in EDTA lysis buffer [50 mM EDTA, 250 mM NaCl, 0.5 mM 4-(2-hydroxyethyl)-1-piperazineethanesulfonic acid, 0.5% Igepal, 10% Complete Mini Protease Inhibitor (Roche Diagnostics GmbH, Mannheim, Germany)], incubated for 1 h at 4°C and centrifuged for 15 min (10,000 ***g***, 4°C). Additionally, whole left tibiae were frozen in liquid nitrogen and homogenized using a mixer mill (MM400, Retsch GmbH, Haan, Germany) and lysed in buffer according to a previously described protocol (Kovtun et al., 2016). Total protein concentrations were determined using the Bicinchoninic Acid Protein Assay Kit (Thermo Scientific, Rockford, USA). Western blotting was performed as described previously (Uschold-Schmidt et al., 2012; Füchsl et al., 2013). Equal amounts of protein lysates (adrenal glands: 15 µg; tibiae: 20 µg) were loaded onto 10% sodium dodecyl sulfate polyacrylamide gels and subsequently transferred to nitrocellulose membranes (Amersham Protran Premium 0.45 µm NC, GE Healthcare Life Science, Freiburg, Germany). The following antibodies were used: primary rabbit anti-TH antibody [adrenal glands: 1:12,000, 1 h, room temperature (RT); tibiae: 1:1000, overnight, 4°C; AB152, Merck KGaG, Darmstadt, Germany]; HRP-conjugated goat anti-rabbit antibody (1:3000, Cell Signaling Technology, New England Biolabs GmbH, Frankfurt am Main, Germany); primary mouse anti-glyceraldehyde 3-phosphate dehydrogenase (GAPDH) antibody (adrenal glands: 1:5000; tibiae: 1:1000; 4°C; Thermo Fisher Scientific, Waltham, USA), HRP-conjugated horse anti-mouse antibody (adrenal glands: 1:10,000, 30 min; tibiae: 1:3000, 1 h; RT, Cell Signaling Technology). Semiquantitative densitometric analyses of the signals were performed using Image Lab™ Software (Bio-Rad Laboratories GmbH, Munich, Germany). TH (∼62 kDa) protein expression for each mouse was normalized to GAPDH (∼36 kDa) expression and averaged by group.

### qPCR

Distal right tibiae were cut and homogenized according to the protocol for whole left tibiae as described above. Total RNA was isolated using the trizol/chloroform method as described previously (Wehrle et al., 2015). A total of 1 µg of isolated RNA was transcribed into cDNA using the Omniscript Reverse Transcriptase Kit (Qiagen, Hilden, Germany). qPCR was performed using the Brilliant Sybr Green QPCR Master Mix Kit (Stratagene, Amsterdam, The Netherlands) according to the manufacturer's protocol in a total volume of 25 µl using the following cycling conditions: 50°C for 2 min, 95°C for 2 min, 40 cycles each consisting of 95°C for 15 s and 60°C for 1 min. Then, melting-curve acquisition was performed (95°C for 15 s, 60°C for 1 min, 95°C for 15 s). *Gapdh* was used as the housekeeping gene (F: 5′-CACTGAATTCACCCCCACTGA-3′, R: 5′-TCTCGATCCGTACAGACGGT-3′). The primers for osteocalcin (*Bglap*) were F: 5′-GCGCTCTGTCTCTCTGACCT-3′ and R: 5′-ACCTTATTGCCCTCCTGCTT-3′, for *Runx2* F: 5′-CCACCACTCACTACCACACG-3′ and R: 5′-CACTCTGGCTTTGGGAAGAG-3′ and for *TH* F: 5′-AACCCTCCTCACTGTCTCGGGC-3′ and R: 5′-TCAGACACCCGACGCACAGAACT-3′. Relative gene expression was calculated using the delta-delta CT method with PCR-efficiency correction using LinRegPCR software as described previously (Ramakers et al., 2003).

### Statistics

Sample size was calculated based on preliminary results from this study with an effect size of 1.37 and power: 80%, alpha=0.05 (G*Power software, free software from Düsseldorf University, Germany). The results of the present study were analyzed for normal distribution using the Shapiro–Wilk test, and significance was tested using the Mann–Whitney *U*-test for single comparisons. All results are presented in the figures as individual dot plots with the median value marked in red and the range in black. Values of *P*≤0.05 were considered to be statistically significant: *0.05≥*P*≥0.01, **0.01>*P*>0.001 and ***P<0.001. Statistical analysis was performed using GraphPad Prism software (version 7).

## Supplementary Material

Supplementary information

First Person interview
