# Supplementary material for: Chronic psychosocial stress disturbs long-bone growth in adolescent mice
Source: Dis Model Mech. 2017 Dec 1;10(12):1399–409. doi: 10.1242/dmm.030916 (PMC5769608; doi:10.1242/dmm.030916)
Supplement: Supplementary information [file dmm-10-030916-s1.pdf]

Supplemental material

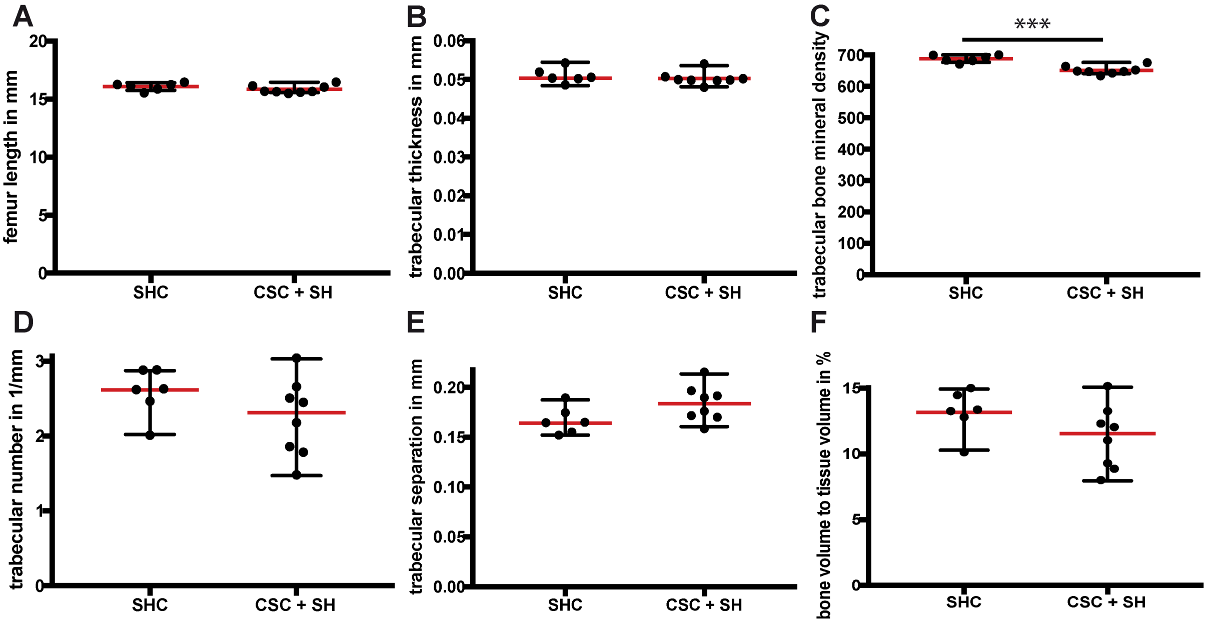

Supplemental Fig. S1: Trabecular bone parameters and length of the femur following 19 days of chronic subordinate colony housing (CSC) and 21 days of single housing. Controls were single housed for 41 days. A) Length of the femur, B) trabecular thickness, C) trabecular bone mineral density, D) trabecular number, E) trabecular separation, F) bone volume/total volume ratio. SHC=single-housed control. Data are displayed as individual dot plots with median ± range.

Disease Models & Mechanisms • Supplementary information

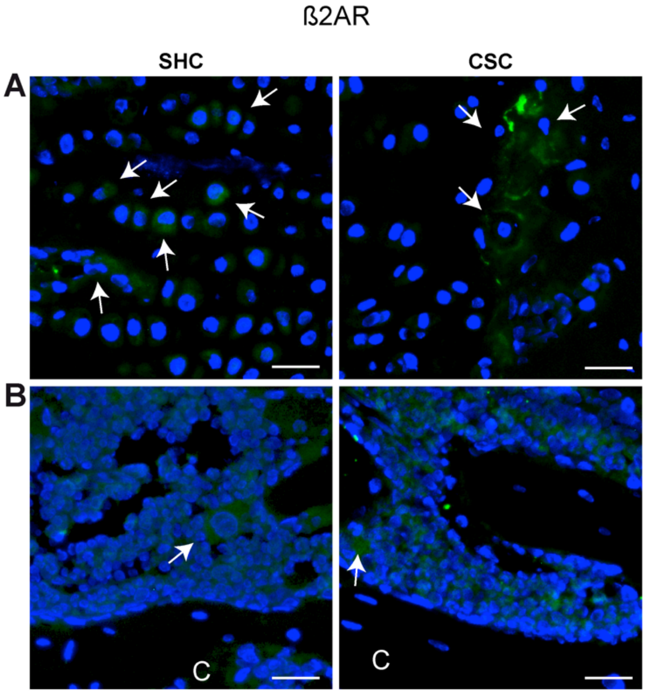

Supplemental Fig. S2: Sympathetic signaling in the femur following 19 days of chronic subordinate colony housing (CSC). A) β2-adrenergic receptor (β2AR) staining at the growth plate and B) the metaphyseal region (cortex=C) of the femur. Scale bar: 25 µm, n=4.
