# Supplementary material for: Chronic psychosocial stress disturbs long-bone growth in adolescent mice
Source: Dis Model Mech. 2017 Dec 1;10(12):1399–409. doi: 10.1242/dmm.030916 (PMC5769608; doi:10.1242/dmm.030916)
Supplement: First Person interview [file dmm-10-030916-s2.pdf]

© 2017. Published by The Company of Biologists Ltd | Disease Models & Mechanisms (2017): doi:10.1242/dmm.030916: Supplementary information

FIRST PERSON

First person – Sandra Förtsch

First Person is a series of interviews with the first authors of a selection of papers published in Disease Models & Mechanisms, helping early-career researchers promote themselves alongside their papers. Sandra Förtsch is first author on ‘Chronic psychosocial stress disturbs long-bone growth in adolescent mice’, published in DMM. Sandra is a PhD student in the lab of Stefan O. Reber at the University Clinic Ulm, Germany, investigating the effects of chronic psychosocial stress on behaviour, inflammation and physiological parameters.

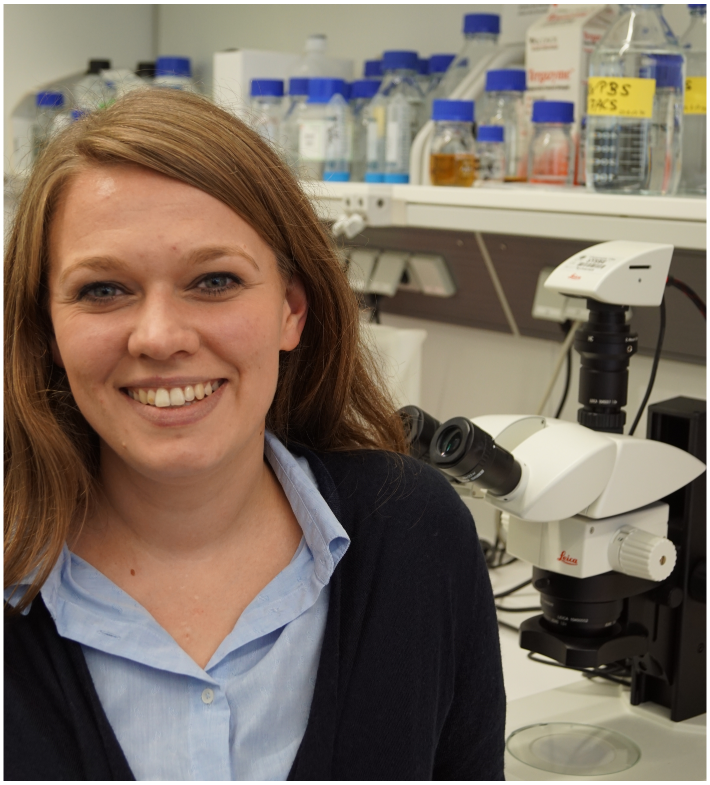

Sandra Förtsch

How would you explain the main findings of your paper to non-scientific family and friends?

Stress – almost everybody is familiar with this term and everybody experiences multiple, more or less, stressful situations during life. When such a stressful situation lasts for weeks or months and is caused by the fact that we are living in a social environment, we are talking about ‘chronic psychosocial stress’. This is a risk factor for the development of a variety of diseases, ranging from mood disorders, e.g. anxiety disorders, posttraumatic stress disorder (PTSD) or depression, to somatic disorders, e.g. cardiovascular and chronic inflammatory disorders, only to name a few. Both depression and PTSD are associated with bone loss and an increased bone fracture risk. In depressed patients this is thought to be due to increased levels of glucocorticoids, whereas the mechanisms in PTSD patients still remain to be elucidated. In this study, we collaborated with Anita Ignatius’ group (Institute of Orthopedic Research and Biomechanics, University Medical Center Ulm), and particularly with Melanie Haffner-Luntzer, to investigate whether chronic psychosocial stress influences bone metabolism and how this could be mediated. To induce chronic psychosocial stress, we used the chronic subordinate colony housing (CSC) paradigm, which takes advantage of the facts that male mice instinctively establish social hierarchies when group-housed and that subordination within such a social hierarchy poses a chronic psychosocial stressor for respective individuals. Besides typical physiological stress consequences, like increased adrenal and decreased thymus weight, our data revealed increased anxiety-related behaviour and disturbed long bone development in chronically stressed compared with unstressed control mice, which were individually housed in their home cages. The long bones of the stressed mice were significantly shorter, and this was accompanied by an increased growth plate width, likely due to a disturbed chondrocyte differentiation. As one possible underlying mechanism, our data suggest increased signalling of certain neurotransmitters, induced by chronic stress locally in the bone.

What are the potential implications of these results for your field of research?

By providing an experimental mouse model for stress-induced disturbances of long bone metabolism and a possible mechanistic explanation, our data sets the stage for future mechanistic experiments contributing to a better understanding of the complex interactions between psychological or psychosocial factors and the development of stress-associated somatic pathologies, e.g. the increased fracture risk in depressed patients.

“I also believe that early-career scientists benefit greatly from interdisciplinary collaborations.”

What are the main advantages and drawbacks of the model system you have used as it relates to the disease you are investigating?

As the CSC paradigm represents a mouse model of chronic psychosocial stress, it strongly resembles the type of health-compromising stress that humans face during everyday life. Thus, it is not surprising that in line with what has been reported in chronically stressed humans, the CSC model increases general and social anxiety-related behaviour, enhances ethanol consumption and preference, causes development of spontaneous colitis and chronic low-grade inflammation, enhances the cancer risk and reduces the diversity of the gut microbiome. Thus, the CSC paradigm is a promising model to study the complex behaviour-neuro-endocrine-immune interactions underlying several mental and somatic stress-associated disorders. However, although we showed a negative effect on bone growth after

Sandra Förtsch’s contact details: Laboratory for Molecular Psychosomatics, Clinic for Psychosomatic Medicine and Psychotherapy, University Ulm, D-89081 Ulm, Germany.

E-mail: sandra.foertsch@uni-ulm.de

19 days of CSC exposure, it is still not clear whether these changes might contribute to an increased risk for bone loss or bone fracture as it is reported in depressed patients. And we still have to keep in mind that the CSC paradigm is a good tool to study chronic psychosocial stress in mice, but yet we are not sure if these results generally can be translated to the human situation.

What changes do you think could improve the professional lives of early-career scientists?

International graduate school programs are a very good opportunity for early-career scientists. The workshops, meetings and symposia organized by graduate schools are a good chance to train yourself in presenting your data. I also believe that early-career scientists benefit greatly from interdisciplinary collaborations. At this stage of their careers the final research direction is still flexible and, therefore, as many experiences as possible should be collected in various fields of research.

What's next for you?

In terms of this project, we will focus on unravelling the underlying mechanisms. For me personally, my next goal is to finalize my PhD thesis and then to find an interesting and challenging postdoctoral position.

Reference

Föertsch, S., Haffner-Luntzer, M., Kroner, J., Gross, F., Kaiser, K., Erber, M., Reber, S. O. and Ignatius, A. (2017). Chronic psychosocial stress disturbs long-bone growth in adolescent mice. *Dis. Model. Mech.* **10**, doi:10.1242/dmm.030916.
